# Experimental Study on the Crack Concrete Repaired via Enzyme-Induced Calcium Carbonate Precipitation (EICP)

**DOI:** 10.3390/ma17133205

**Published:** 2024-07-01

**Authors:** Gang Li, Deqiang Yan, Jia Liu, Peidong Yang, Jinli Zhang

**Affiliations:** 1 Shaanxi Key Laboratory of Safety and Durability of Concrete Structures, Xijing University, Xi’an 710123, China; t_bag945@126.com (G.L.); 19915445726@163.com (D.Y.); yang_peidong2023@126.com (P.Y.); 2 State Key Laboratory of Coastal and Offshore Engineering, Dalian University of Technology, Dalian 116024, China; jlzhang@dlut.edu.cn

**Keywords:** concrete, EICP, crack repair, compressive strength, microscopic mechanism

## Abstract

A low-carbon and environmentally friendly EICP method for repairing concrete cracks is presented to prolong the service life of concrete. In this study, we took concrete as the research object and quartz sand as the filling medium and employed the EICP injection method to repair concrete cracks. The internal repair effect of EICP on concrete cracks was evaluated with a combination of ultrasonic and compressive strength tests. The concrete repair mechanism of EICP was identified with a combination of EDS, XRD, and SEM tests. The results indicate that with an increase in the fracture depth, the ultrasonic sound time of the crack specimen increased gradually, and the ultrasonic wave transit time value of the crack specimen decreased significantly after EICP repair. After repair, the compressive strength rose. The highest compressive-strength recovery rate of a 0.3 mm wide specimen is 98.41%. The calcium carbonate crystal formed using EICP is vaterite. The probability density function model of the Laplace distribution was constructed, which showed good applicability and consistency in the ultrasonic sound time and compressive strength measured via experiments. The formed calcium carbonate crystals can be tightly and evenly attached to the cracks with the EICP injection repair method, resulting in a better repair effect.

## 1. Introduction

**Figure 1:**
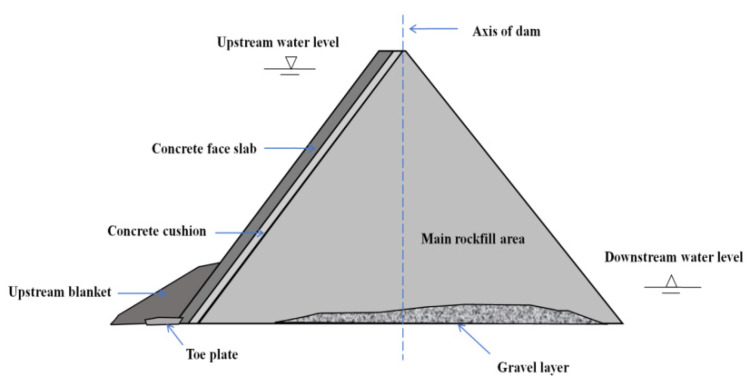
Profile of concrete-face rockfill dam.

Earth–rockfill dams are the fastest growing type in dam construction worldwide. It is widely used and developed because of its high economy, ability to be made with local materials, and suitability for all kinds of operating conditions. The concrete-face rockfill dam uses the concrete face on the upstream surface as the anti-seepage structure, as shown in Figure 1. As an earth–rock dam, it has been commonly used because of its good permeability, seismic performance, and slope stability [1]. Micro-cracks are produced in the face dam with water pressure, freeze–thaw, carbonization, and other intervention methods [2]. Traditional crack repair methods, such as the surface repair, grouting, and filling methods, are suitable for cracks with small depths [3], and the grouting and filling methods are suitable for deep cracks, but the grouting material pollutes the environment [4,5]. The repair method of an impermeable wall can be adopted for deep and significant cracks, but the cost is high, it takes a long time, and the construction process has certain risks. Although the above-mentioned crack repair methods have been applied, each method still has certain problems. Therefore, it is necessary to develop an efficient and environmentally friendly concrete crack repair method in laboratory research and ultimately use it in on-site crack repair experiments.

The principle of enzyme-induced calcium carbonate precipitation (EICP) technology is catalyzing the hydrolysis of urea into carbonate ions using plant enzymes and then forming calcium carbonate precipitation and cement soil particles with the introduced calcium source to solidify the soil. EICP technology for measuring nanometer-level urease sizes, suitable for fine particle cementation, has the advantages of low cost, high compatibility, easy operation, environmental friendliness, and broad research prospects [6,7,8,9]. It is widely used in soil seepage control [10], foundation improvement [11], contaminated soil remediation [6], etc. Song et al. [12] aimed to inhibit the production of particulate matter (PM2.5 and PM10) from wind and dynamic effects to solve the environmental and sustainability problems caused by dust and traditional emission reduction. Their results showed that, when the volume of the EICP solution was increased, the shear wave velocity and peak cone tip persistence almost doubled, which can effectively reduce dust emesis. Rahman et al. [13] conducted EICP treatment on different oil types via electrical conductivity, unconfined comprehensive strength, and erosion resistance tests and found that the mass loss rate and strength of calcium carbonate show a trend of decline with increases in the number of dry–wet and freeze–thaw cycles and temperature. Pisani et al. [14] used the density functional theory (DFT) to study the consolidation effect of four common dry soil components after EICP was used to produce a DFT simulation containing amino acids. The results indicated that urease could bind to various oxides in arid oil with alanine and glycine. Lin et al. [15] treated the surrounding soil–cement using several concrete model piles, and the results showed that EICP treatment could form soil–cement belts around several concrete piles, which improved the bearing and load transfer capacities of these piles under comprehensive loads. Wu et al. [16] studied the influence of EICP on wind-blown sand consolidation under different PVA concentrations via unconfined comprehensive strength, wind erosion, water erosion, and permeability persistence tests. The results showed that the presence of PVA results in improvements in the unconfined comprehensive strength, wind erosion resistance, water erosion resistance, and surface strength of EICP consolidated sand. Moghal et al. [17] studied the adsorption and desorption capacities of cadmium (Cd), nickel (Ni), and other bivalent heavy metals in tropical soil. The results showed that the adsorption values of Cd and Ni after EICP treatment were 3.457 and 4.418 mg/g, respectively, indicating that the effect of EICP treatment on soil contaminated by heavy metal ions was significant. He et al. [18] used the EICP method to assess the performance of silty sand through a soil model penetration test and a triaxial CD test. The results showed that the osmotic resistance in the model increased with an increase in processing time. The penetration resistance distribution is also consistent with the distribution of calcium carbonate content in the model. Miao et al. [19] generated a field test on hardened desert sand using an innovative method combining EICP and polyacrylamide (PAM). The results showed that the sclerosis strength of EICP + PAM is 6.0~7.0% higher than that of EICP alone under the same conditions. This method has high persistence in high wind erosion conditions.

The above studies show that EICP technology has achieved good results in dust reduction, soil solidification, improving concrete pile-bearing capacity, and wind sand induration. The repair effect of EICP technology on concrete cracks, the mechanical properties and microscale EICP repair mechanism after repair, and the relevant numerical simulation construction have been rarely studied by scholars. Accordingly, this study took concrete as the research object and quartz sand as the repair medium and adopted the EICP injection method to repair concrete cracks. The ultrasonic sound time and compressive strength recovery rate after EICP repair were determined. The chemical composition, crystal morphology, and material morphology of EICP were analyzed via EDS, XRD, and SEM tests, and the EICP repair mechanism was observed. The probability density function model of the Laplace distribution was established. The research results can provide a reference for the practical engineering application of crack repair in concrete-face rockfill dams.

## 2. Materials and Methods

### 2.1. Test Materials

PO42.5 ordinary Portland cement (Taiyuan Shanshui Cement Co., Ltd., Taiyuan, China) and Grade II fly ash (Rongchangsheng Environmental Protection Materials Co., Ltd., Suzhou, China) were used in the test. According to the standard method for the chemical analysis of cement (GB/T 176-2017) [20], the chemical compositions of cement and fly ash are shown in Table 1. Crushed stones with a density of 2700 kg/m^3^ and 5~20 mm and natural river sand with a density of 2698 kg/m^3^ and a fineness modulus of 2.74 were selected as the aggregates. Polycarboxylic acid is a high-performance water-reducing agent with a water-reducing rate of 45% (Tianjin Feilong Concrete Admixture Co., Ltd., Tianjin, China). The design strength grade of test concrete is C30, the water–cement ratio is 0.52, and the mix ratio is shown in Table 2. Commercial soybeans were used as a urease source, and were produced in Heilongjiang. The test reagents included urea (Tianjin Hengxing Chemical Reagent Manufacturing Co., Ltd., Tianjin, China) and calcium chloride (Sinopharm Chemical Reagent Co., Ltd., Shanghai, China), all of which were analytical pure reagents.

### 2.2. Sample Preparation

**Figure 2:**
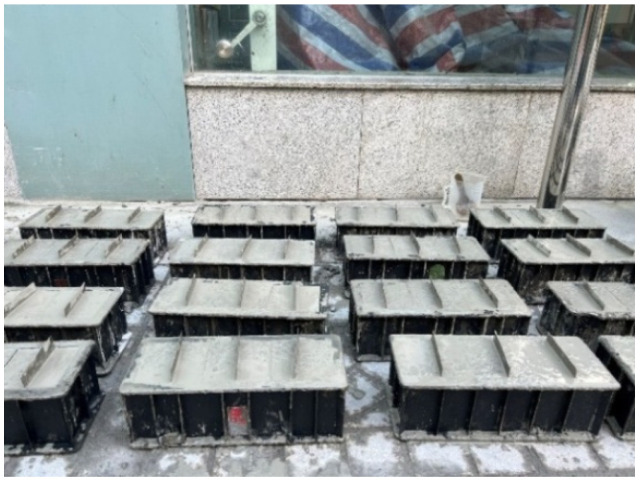
Precast concrete cracks using steel plate insertion method.

Concrete is prepared according to the standard for test methods of the mechanical properties of ordinary concrete (GB/T 50081-2019) [21]. Cement, fly ash, aggregate, water reducer, and water are weighed according to the concrete mix ratio shown in Table 2 and mixed in an HJS-60 double horizontal shaft concrete mixer (Tianjin Gangyuan Test Instrument Factory, Tianjin, China) at Shaanxi Key Laboratory for 1–3 min. Then, the mixed mixture is put into a 100 × 100 × 100 mm mold brushed with the release agent, steel sheets are inserted in the middle of each concrete test block, and the mixture is vibrated on a mechanical shaking table (Tianjin Gangyuan Test Instrument Factory) for 30–60 s. After this process is complete, the excess mixture is scraped off with a mud wiping knife, the abrasive tools and steel sheets are removed after 24 h, and the cracks are repaired after curing in the YH-40B standard constant temperature and humidity curing box (Tianjin Gangyuan Test Instrument Factory, Tianjin, China) for 28 days. The concrete samples are prepared as shown in Figure 2.

**Figure 3:**
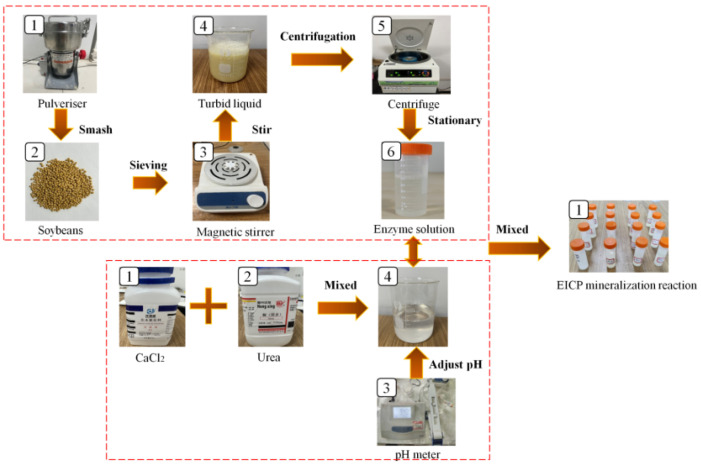
Preparation process of the enzyme and cement solutions.

Fresh soybeans were crushed for 5 to 10 min in an 800 A crusher (manufactured by Yongkang Jinwei Electric Appliance Co., Ltd., Yongkang, China) and filtered using a 100-mesh sieve to collect soybean powder. Soybean flour was added to deionized water, stirred with an SN-MS-1 magnetic stirrer (manufactured by Xi’an KTL Instruments Co., Ltd., Xi’an, China) for 1 h, and kept still for 24 h. Then, the solution was placed into a TGL-16G centrifuge (manufactured by Hunan Kecheng Instrument and Equipment Co., Ltd., Changsha, China) and centrifuged for 15 min at 4 °C and 3000 r/min to extract the supernatant solution and obtain the enzyme solution. Calcium chloride and a urea solution were used to prepare the cementing fluid. The pH values of the two solutions were adjusted with a pH meter, and the crystal form was stabilized by adding skim milk powder. The enzyme solution was mixed with the cement solution and catalyzed the hydrolysis of urea in the cement solution to obtain carbonate and ammonium ions. The calcium ions in the cement solution reacted with the hydrolyzed carbonate ions to form calcium carbonate precipitates, which were used for repairing mortar cracks. Previous experiments considered the effects of the incorporation ratio and concentration of the enzyme solution, pH value, and the content of skim milk powder on the EICP reaction rate. Previously, tube tests were used to obtain the optimal EICP mineralization reaction conditions and consider the effects of the incorporation ratio and concentration of the enzyme solution, pH value, and the content of skim milk powder on the EICP reaction rate. Tube tests obtained the following optimal factors: an enzyme solution concentration of 100 g/L; a cement fluid concentration of 1.0 mol/L; a skim milk powder content of 6 g/L; a pH of 7.0; and an enzyme incorporation ratio of 0.1 (see Appendix A). Figure 3 shows the preparation processes of the enzyme and cement solutions.

### 2.3. Test Method

The EICP injection method was used to repair concrete cracks [22]. After concrete curing was complete, 60% quartz sand was uniformly added to the cracks as the repair medium. Based on the optimal EICP repair conditions from previous experiments, 100 g/L and 1.0 mol/L of the enzyme and cement solutions, respectively, were mixed in a 1:1 volume to create the EICP repair solution. After 30 min, once the reactions reached 20 °C and had a pH of 7.0, the reaction solution was extracted with a syringe and injected into the concrete specimen until the interior was filled with the repair solution. Absorbent cotton balls were placed at both ends of the crack to absorb excess reaction fluid. The repair fluid was injected every 1 h and 8 times a day for 10 days.

The ultrasonic and compressive strength tests were carried out using both an NM-4A nonmetallic ultrasonic detector (Beijing Kangkerui Engineering Inspection Technology Co., Ltd., Beijing, China) and a WAW-1000 microcomputer-controlled electro-hydraulic servo universal testing machine (MTS Company, Eden Prairie, MN, USA) after the concrete test block was repaired to describe the effect of repairing concrete cracks via the EICP injection method in a more intuitive manner. The crack depth (*C_d_*) and width (*C_w_*) in concrete were determined using the results of related studies [23], where B0 is the crack-free specimen. The experimental scheme is shown in Table 3. The ultrasonic sound time (*r*_1_) and compressive strength loss rate (*r*_2_) are measured according to Formulas (1) and (2), respectively.

(1) $$r_{1}=\frac{t_{1}-t_{0}}{t_{0}}\times 100\%$$

where *t*_1_ and *t*_0_ are the ultrasonic sound times at crack depths of 35 to 65 mm and 20 mm, μs, respectively.

(2) $$r_{2}=\frac{c_{1}-c_{0}}{c_{0}}\times 100\%$$

where *c*_1_ and *c*_0_ are the compressive strengths of the crack-free and unrepaired specimens, MPa, respectively.

## 3. Results and Analysis

### 3.1. Analysis of Ultrasonic Testing Results of Concrete Test Block

**Figure 4:**
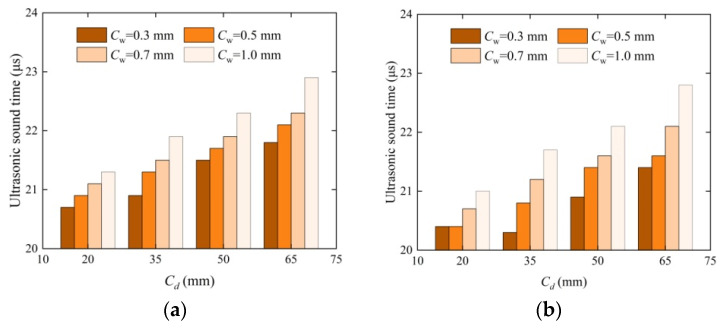
Histogram of changes in ultrasonic sound time before and after repair of concrete with different crack widths: (**a**) unrepaired and (**b**) repaired test blocks.

Ultrasonic crack detection technology can reflect the repair effect of concrete cracks to a certain extent. Figure 4 shows a histogram of the ultrasonic sound time changing with depth before and after the concrete repair of four crack widths. The ultrasonic acoustic time value of the cracked test block is higher than the nondestructive test block, the ultrasonic sound time of the test concrete specimen repaired using EICP is reduced, and the peak diffuse transmission time is slower than that of the unrepaired test block, as shown in Figure 4. As the crack width increases, the loss of ultrasonic sound time is significant, which is consistent with the reference research. As the fracture depth increases, the acoustic time loss of the crack block increases. Among them, the average losses of the acoustic time values of 20 mm and 65 mm crack depth are smaller and larger, respectively. As the crack depth increased, the loss rate of the acoustic time value gradually increased. At a crack width of 0.3 mm, the ultrasonic sound time at a crack depth of 20 mm is 20.3 μs, and the ultrasonic sound time at a crack depth of 65 mm is 21.4 μs. According to Formula (1), the loss rate of the measured ultrasonic sound time (*r*_1_) is 5%, which is not significantly different from the 0.5 mm crack width. The maximum loss rate of sound time at a crack width of 1.0 mm is 8%. Among all test blocks, the minimum loss rate of acoustic time value is 1.5% at a crack depth of 35 mm and a width of 0.3 mm. In a specimen with a 1.0 mm width, the average loss rate is between 5% and 13% at different depths, and the maximum loss rate is 13% at a depth of 65 mm. The main reason for the above phenomenon is that after repairing concrete with different crack widths via EICP injection, the precipitates adhere to the crack surface. Quartz sand has a coarse surface, which can improve the internal filling effect as a repair medium. The loss rate of the sound time value with a smaller crack width is better than that with a larger crack width. As the crack depth increased, the internal defects increased, and the distance of ultrasonic acoustic emission grew longer.

### 3.2. Analysis of Test Results of Compressive Strength of Concrete Test Block

**Figure 5:**
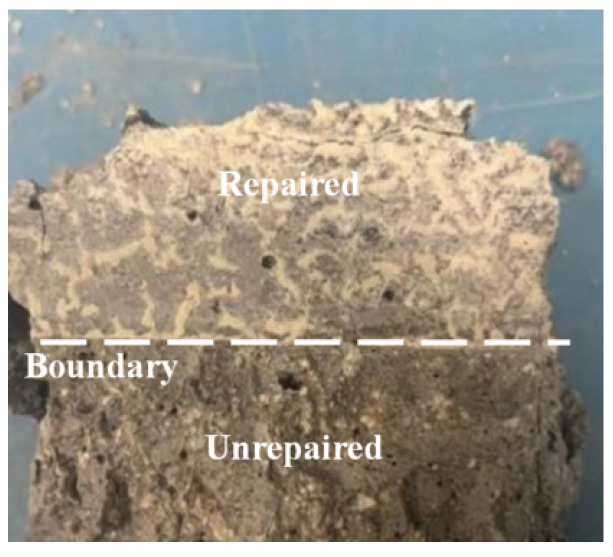
The sample after compression test.

**Figure 6:**
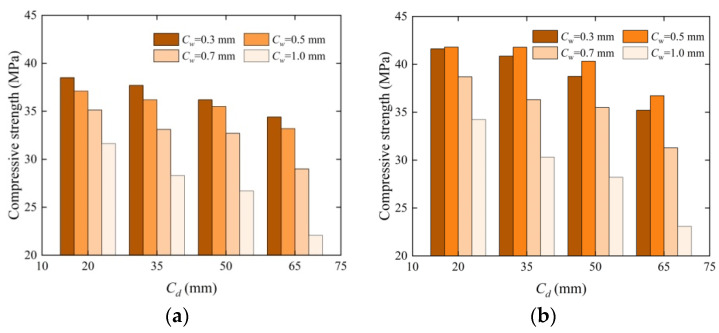
Histogram of compressive strength of concrete before and after repair with depth under different crack widths: (**a**) unrepaired and (**b**) repaired test blocks.

**Figure 7:**
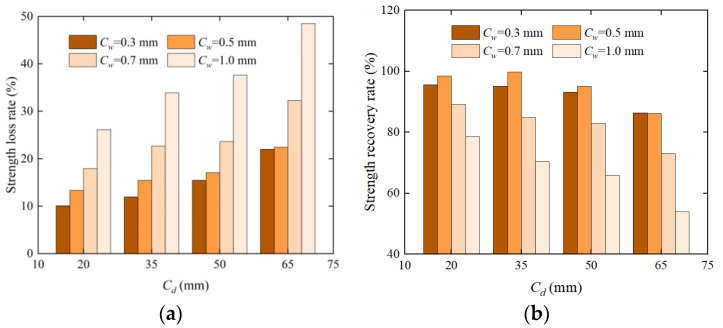
Histogram of strength loss and recovery rates of specimens with different crack widths before and after repair as a function of crack depth: (**a**) unrepaired and (**b**) repaired test blocks.

Compressive strength has an excellent characterization effect on the internal defects of concrete [24], which can reflect the repair effect of EICP on concrete. Figure 5 shows the sample image of the compression test. Figure 6 shows a histogram of the compressive strength and crack depth of concrete specimens under four crack widths, and Figure 7 shows a histogram of compressive strength loss rate and recovery rate changing with crack depth under four crack widths. These figures show that after the compression test, the upper boundary of the concrete test block is covered with a large amount of white material on the surface, including quartz sand and calcium carbonate. As the crack width and depth increased, the compressive strength decreased. The average strength loss rate of the specimen with a 0.3 mm width is the lowest, and the minimum quality loss rate of concrete specimens under a crack depth of 20 mm is 10.05%, which is only 0.38~0.75 times higher than the strength loss of other specimens and has little difference from the strength loss rate of the specimen with a 0.5 mm width and different crack depths. The average loss rate of compressive strength of the specimen with a 1.0 mm crack width is the highest, and the maximum compressive strength loss rate for a crack depth of 65 mm is 48.48%, which is 4.82 times the strength loss of the specimen with the smallest width. The compressive strength of concrete blocks repaired using EICP is significantly higher than that of unrepaired concrete blocks. The highest recovery rate of compressive strength of a 0.3 mm width specimen is 98.41%, and the lowest recovery rate of compressive strength of a 1.0 mm width specimen is 53.93%. This is consistent with the results of ultrasonic testing because the cracks treated using EICP were attached and wrapped due to calcium carbonate precipitation, like a new “skin” on the crack surface. Quartz sand, as a repair medium, is combined with EICP, which improves the compressive strength and reflects the feasibility of EICP to repair concrete more intuitively. As the crack width and depth increased, the “skin” gradually expanded, the calcium carbonate generated in the test sample was loose, and its cementing performance was not fully displayed, so the test concrete specimen was more prone to compression damage.

### 3.3. Analysis of the Effect of Fracture Size on Calcium Carbonate Formation Efficiency

Calcium carbonate deposits formed in the fracture after EICP repair can directly reflect the repair effect of EICP. A fracture depth of 65 mm was selected, and splitting was carried out according to different depths. The calcium carbonate formation within 5 mm of the fracture was measured using the acid washing method [25]. Table 4 shows the formation rate of calcium carbonate under four crack widths. As shown in the table, the formation of calcium carbonate increased as the crack width decreased, and it decreased as the crack width increased. The formation rate of calcium carbonate is 79.33% at 0.5 mm and 58.33% at 1.0 mm, which is consistent with the above analysis results.

**Figure 8:**
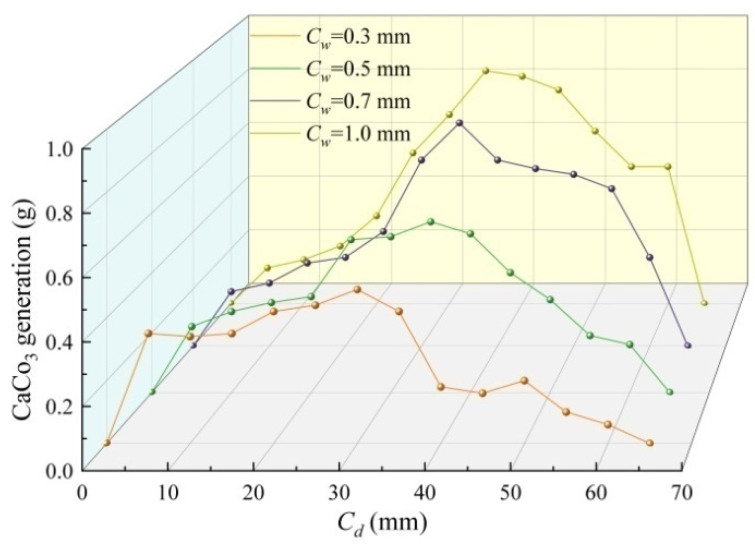
Waterfall plot of calcium carbonate production under different crack widths.

Figure 8 shows a waterfall plot of calcium carbonate production varying with fracture depth under four different fracture widths. As shown in the figure, the deposition amount of calcium carbonate will show different aggregation points with an increase in fracture width. For 0.3 mm fractures, calcium carbonate deposits are mainly concentrated within the fracture depth of 5~35 mm, and the cracks fluctuate within 35~65 mm. The overall distribution of calcium carbonate deposits is relatively symmetrical, with a fracture width of 0.5 mm. For 0.7 mm and 1.0 mm fracture widths, the distribution is predominantly concentrated in a crack depth of 30~65 mm and less concentrated in a fracture depth of 0~30 mm. The main reason for the above phenomenon is that as the fracture width increased, the flow field of the repair fluid increased, and calcium carbonate gradually transferred to the middle and lower ends of the fracture. Among them, crack widths of 0.5 mm are more conducive to penetrating the repair fluid into crack depths than crack widths of 0.3 mm, possibly because narrow cracks will limit the fluidity of repair fluid and lead to uneven calcium carbonate generated in cracks.

### 3.4. Mechanism Analysis of EICP Repairing Concrete Blocks

**Figure 9:**
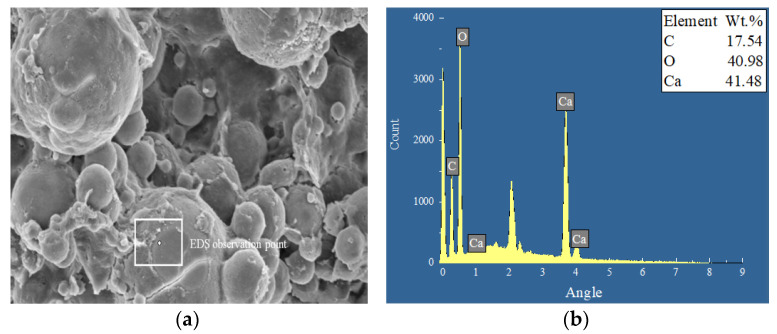
Material morphology and EDS map of concrete cracks repaired by EICP: EDS (**a**) measuring point and (**b**) spectrogram.

**Figure 10:**
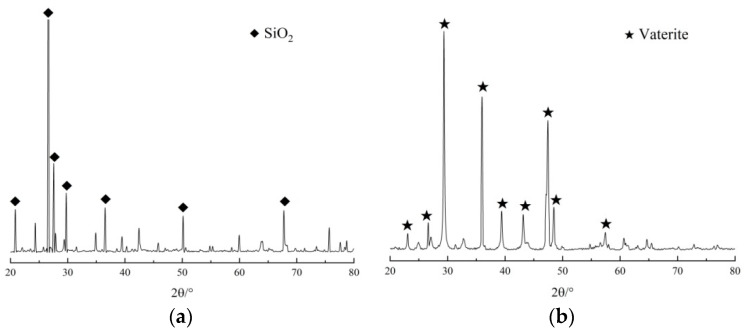
XRD pattern of EICP repaired concrete block: (**a**) unrepaired and (**b**) repaired test blocks.

The phase composition, crystal composition, and microstructure of the repaired concrete test block were analyzed microscopically with a combination of EDS, XRD, and SEM tests to identify the action mechanism of the EICP repairing concrete crack test block [26]. Figure 9 shows the internal material morphology and EDS map of the concrete block repaired using the EICP injection method. Figure 10 shows the XRD pattern of concrete specimens using the EICP injection method. These figures show that the mineral is spherical, and the main elements in the EDS energy spectrum are C, O, and Ca, accounting for 17.54%, 40.98%, and 41.48%, respectively, which proves that the mineral is calcium carbonate. The diffraction peak in the picture is metastable vaterite, as identified with XRD analysis. The unrepaired concrete specimens are mainly SiO_2_, and the XRD diffraction peaks are pronounced. After repair, almost all calcium carbonate crystals produced using EICP were vaterite, the corresponding peaks were relatively reduced, the crystal characteristic peaks were sharp, and the crystallinity was fine. CaCO_3_ crystals formed on the surface can facilitate the mineralization and fixation of concrete components.

**Figure 11:**
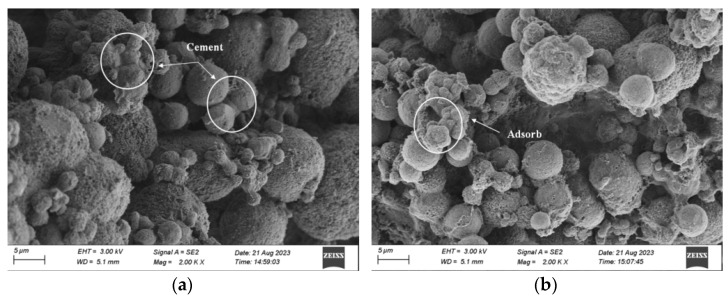

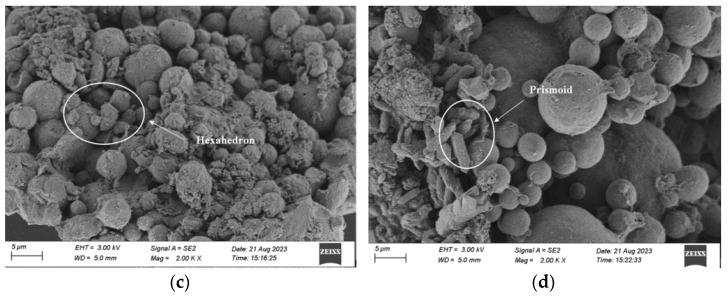
Microstructure diagram of EICP repaired concrete block under four crack widths: (**a**) *C_w_* = 0.3 mm; (**b**) *C_w_* = 0.5 mm; (**c**) *C_w_* = 0.7 mm; and (**d**) *C_w_* = 1.0 mm.

Figure 11 shows the microstructure diagram of the EICP-repaired concrete block with four crack widths. As shown in the graph, the calcium carbonate crystals generated via EICP are distributed inside the cracks, and the calcium carbonate crystals with crack widths of 0.3 mm and 0.5 mm are mainly spherical. From the above analysis, we concluded that the spherical crystals were vaterite. The distribution of calcium carbonate crystals is uniform under crack widths of 0.3 mm and 0.5 mm. Adjacent particles become whole via cementation and adsorption, and the contact between particles is surface contact. The surface roughness of concrete and calcium carbonate crystals increased by including the quartz sand repair medium, and their cohesion improved. The surface of large crystal particles was covered with fine crystals, and the crystal structure was close. At crack widths of 0.7 mm and 1.0 mm, there were not only vaterite but also incomplete hexahedral and prismatic crystals. The distribution of the calcium carbonate formed on the crack surface was uneven, and their cementation and adsorption were low. The results indicate that the formation rate of the calcium carbonate precipitate was affected by the higher crack width, which leads to different crystal shapes and uneven internal dispersion. Therefore, we concluded that the calcium carbonate precipitate can be absorbed by using quartz sand as a repair medium and employing the EICP repair method, and the injection method can make the repair solution enter the crack more smoothly without blocking the surface, making the formed calcium carbonate crystals tightly and uniformly adhere to the cracks, thereby achieving the repair effect of concrete cracks. Overall, EICP technology is promising for research on concrete crack repair.

### 3.5. Establishment and Verification of Laplace Distribution Model

The Laplace distribution probability density function was used to establish a model for repairing concrete cracks using EICP. After repairing with EICP technology, the repair solution could not be injected into the concrete, but there were still tiny pores in the concrete. Assuming that the pores inside the cracks are filled, the repair rate P of concrete can be obtained as follows:

(3) $$P=\frac{V-V_{0}}{V}$$

(4) $$V=l^{3}$$

(5) $$V_{0}=l\times C_{w}\times C_{d}$$

where *V* is the volume of concrete, mm^3^; *V*_0_ is the internal volume of concrete cracks, mm^3^; *l* is the width of the concrete test block, mm; *C_w_* is the crack width, mm; and *C_d_* is the crack depth, mm. To obtain the relationship between the ultrasonic sound time, compressive strength, splitting tensile strength, and crack repair rate *P*, the Laplace distribution function is then established as follows:

(6) $$F\left(x,a,b\right)=\int \left[-\infty ,x\right]f(t,a,b)dt$$

where *a* is the position parameter of ultrasonic sound time and compressive strength, *b* is the scale parameter controlling the distribution amplitude, and *f* (*t*, *a*, *b*) is the probability density function. Then, the Laplace distribution function *F* (*x*, *a*, *b*) defines the distribution function as follows:

(7) $$F\left(x,a,b\right)=\int \left[-\infty ,x\right]\frac{1}{2b}e^{\frac{-t-a}{b}}dt$$

When *t* is less than *a*, Equation (7) can be written as follows:

(8) $$\int \left[-\infty ,x\right]\frac{1}{2b}e^{\frac{t-a}{b}}dt=\left[0,x\right]\frac{1}{2b}e^{\frac{t-a}{b}}+c_{1}=\frac{1}{2b}e^{\frac{x-a}{b}}+c_{1}$$

When *t* is greater than *a*, Equation (7) can be written as follows:

(9) $$\int \left[-\infty ,x\right]\frac{1}{2b}e^{\frac{-(t-a)}{b}}dt=\left[-\infty ,x\right]\frac{1}{2b}e^{\frac{t-a}{b}}+c_{2}=\frac{1}{2b}e^{\frac{a-x}{b}}+c_{2}$$

The probability density function is in the form of an absolute value function, so the probability density function of the Laplace distribution is obtained as follows

(10) $$F\left(x,a,b\right)=\frac{1}{2b}e^{-\frac{\left|x-a\right|}{b}}+c$$

**Figure 12:**
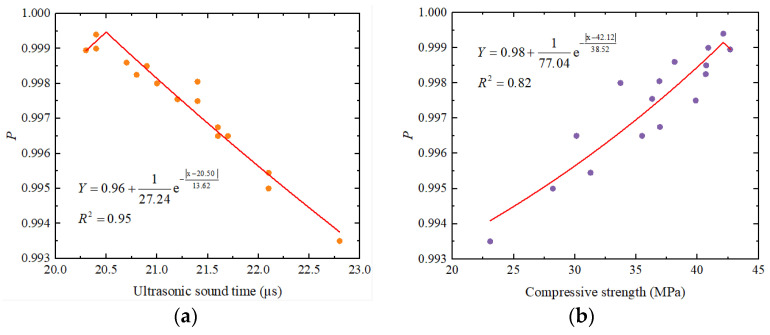
The Laplace distribution function fitting curve of concrete specimens repaired by using EICP: (**a**) ultrasonic sound time and (**b**) compressive strength.

Equation (10) is fitted with the parameters of ultrasonic sound time and compressive strength obtained from the above tests, and the relationship curve between the ultrasonic sound time, compressive strength, and crack repair rate is shown in Figure 12. As shown in the figure, as the crack repair rate increases, there is a negative correlation between ultrasonic sound time and compressive strength, i.e., it will decrease, and the compressive strength will gradually increase, which is consistent with the above analysis. The fitting curves obtained using the probability density function of the Laplace distribution are in good agreement with the ultrasonic time and compressive strength measured in the experiments, in which the fitting degree *R*^2^ of ultrasonic time and compressive strength is 0.95 and 0.82, respectively. Therefore, we concluded that our Laplace distribution probability density model has good applicability and can accurately simulate the variation in the ultrasonic sound time and compressive strength of concrete repaired using EICP with a crack repair rate. However, the deviation in individual test data was caused by the non-uniformity of EICP concrete crack repair, which led to internal and strength deviations in the ultrasonic testing concrete crack test block. This may also be caused by the change in internal pores that may occur during concrete test block vibration.

## 4. Conclusions

In this study, we took concrete as the research object and quartz sand as the filling medium and employed the EICP injection method to repair concrete cracks. We also used ultrasonic and compressive strength tests to evaluate the internal repair effect and strength recovery rate of EICP on concrete cracks and established and verified a Laplace distribution probability density function model. Moreover, the EICP repair mechanism was identified using a combination of EDS, XRD, and SEM tests and a microscopic analysis of concrete specimen material morphology, diffraction pattern, and microstructure. The main conclusions are as follows:

1. After EICP repair, the ultrasonic sound time and peak diffuse transmission time of the specimen decreased significantly. Among all test specimens, the minimum loss rate of acoustic time value was 1.5% at a crack depth of 35 mm and a width of 0.3 mm. The acoustic time loss of the crack specimen increased as the fracture depth increased. The effective repair depth of the fracture repair dividing line was uneven, but it was evenly distributed in a 0.5 mm area.
2. The compressive strength decreased as the crack size increased. The average loss rate of compressive strength of the specimen with a 1.0 mm width was the highest, which was 4.82 times the strength loss of the specimen with the smallest crack width. The average strength loss rate of a 0.3 mm wide specimen was the lowest. The compressive strength of concrete specimens repaired using EICP was significantly higher than that of unrepaired concrete blocks. The highest compressive strength recovery rate of a 0.3 mm width specimen was 98.41%, and the lowest of a 1.0 mm width specimen was 53.93%.
3. Calcium carbonate deposition showed different aggregation points with an increase in fracture width. The highest formation rate of calcium carbonate was 79.33% at 0.5 mm, and the lowest formation rate was 58.33% at 1.0 mm. For 0.3~0.5 mm fractures, calcium carbonate deposits were mainly concentrated in the fracture depth of 5~40 mm, and fractures with a 0.5 mm width were more favorable than those with a 0.3 mm width for repairing liquid by penetrating deep into fractures.
4. The calcium carbonate produced using EICP was a metastable vaterite with a sharp crystal peak and good crystallinity. The results show that CaCO_3_ crystals produced using EICP could realize the mineralization and fixation of concrete components well. By injecting quartz sand combined with EICP, the repair solution could enter the crack more smoothly, resulting in the formation of calcium carbonate crystals that are tightly and evenly attached to the crack to achieve the repair effect.
5. Using EICP to repair concrete cracks and the Laplace distribution probability density function to establish a model, the obtained fitting curve aligned with the ultrasonic sound time and compressive strength measured in the experiments, and sand has good applicability. However, the deviation in individual test data was caused by the non-uniformity of crack repair in EICP concrete, which leads to internal and strength deviations in the concrete crack test block detected by ultrasonic wave.
6. More repair times and days can obtain more calcium carbonate production to fill the cracks, which can achieve better repair effects in the experiment. Therefore, the procedure described in the experiment was relatively cumbersome. In future research, highly active urease will be applied so that the number and time of experiments can be significantly reduced, which is beneficial for improved applications in practical engineering.

## Figures and Tables

**Table 1:** Chemical compositions of cement and fly ash.

Material Content (%)	CaO	SiO_2_	Al_2_O_3_	Fe_2_O_3_	MgO	SO_3_	K_2_O	CaO
Cement	51.4	25.0	8.26	4.0	3.0	2.2	0.4	51.4
Fly ash	4.6	42.6	36.3	2.0	0.8	0.3	1.8	4.6

**Table 2:** Mix proportions of concrete.

Water Consumption (kg·m^−3^)	Cement (kg·m^−3^)	Sand (kg·m^−3^)	Stone (kg·m^−3^)	Fly Ash (kg·m^−3^)	Water Reducer (kg·m^−3^)	Slump (mm)
161	279	993	917	31	4.65	200

**Table 3:** Test scheme of EICP repairing concrete cracks.

Numbering	*C_d_* (mm)	*C_w_* (mm)	Numbering	*C_d_* (mm)	*C_w_* (mm)
B0	—	—	—	—	—
B1	20	0.3	B9	50	0.3
B2	20	0.5	B10	50	0.5
B3	20	0.7	B11	50	0.7
B4	20	1.0	B12	50	1.0
B5	35	0.3	B13	65	0.3
B6	35	0.5	B14	65	0.5
B7	35	0.7	B15	65	0.7
B8	35	1.0	B16	65	1.0

**Table 4:** The yield and generation of calcium carbonate under different crack widths.

*C_w_* (mm)	Gel Solution Concentration (M)	CaCO_3_ Theory Generation Amount (g)	Average CaCO_3_ Generation Amount (g)	Average CaCO_3_ Generation Rate (%)
0.3	1.0	5.0	3.7	74.3
0.5	1.0	6.5	5.2	79.3
0.7	1.0	8.0	5.3	66.7
1.0	1.0	10.0	5.8	58.3

**Figure A1:**
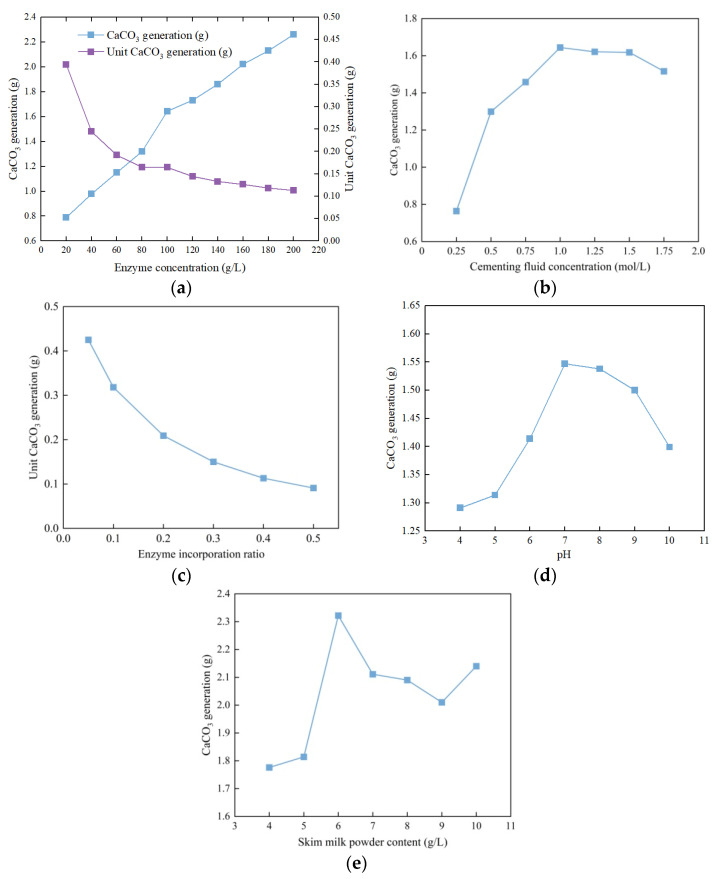
The influence of different influencing factors on calcium carbonate production: (**a**) urease concentration; (**b**) cement fluid concentration; (**c**) enzyme incorporation ratio; (**d**) pH; and (**e**) skim milk powder content.

## Data Availability

Data are contained within the article.

## Appendix A

Figure A1 shows the amount of calcium carbonate generated via EICP technology under different factors using a single factor analysis method. Considering economy and efficiency, the optimal process conditions were determined as follows: enzyme concentration is 100 g/L, cement fluid concentration is 1.0 mol/L, skim milk powder content is 6 g/L, pH is 7.0, and enzyme incorporation ratio is 0.1.
